# Mental health’s shaping influence on college students’ career choices: evidence from L University in Fujian Province, China

**DOI:** 10.3389/fpsyg.2025.1647718

**Published:** 2025-12-16

**Authors:** Cheng Li, Shangping Lin, Xue Deng

**Affiliations:** School of Sociology and Political Science, Anhui University, Hefei, Anhui, China

**Keywords:** career choices, mental health, college students, gender differences, cohort differences

## Abstract

**Objective:**

To investigate the shaping of college students’ mental health on their career choices, considering the influences of gender, year of enrollment, mental health determinants and their interaction on these choices.

**Methods:**

Multinomial logistic regression was used to analyze the mental health and employment data of students at L University in Fujian Province, China. Three models were developed with career choice as the dependent variable. Model 1 comprises solely gender and enrollment year. Model 2 incorporates mental health variables, while Model 3 includes the interaction terms of mental health factors with gender and enrollment year.

**Results:**

Model 1 suggests that men are less inclined to choose female-dominated sectors—such as finance and public services, service industry—and are more likely to pursue self-employment. Students who enrolled in 2018 and graduated during economic recovery had broader employment opportunities than those who enrolled in 2019 and graduated during economic downturn. Model 2 reflects that external stress might compel vocational students to explore alternative career paths. Conversely, severe psychopathology diminishes confidence and restricts opportunities in organized domains such as finance and public services. Social difficulties and romantic distress exemplify occupation-specific inclinations related to interpersonal dynamics. Model 3 shows that gender and enrollment year moderate these effects. For instance, men would address emotional and stress-related challenges through proactive strategies, but the 2019 cohort exhibited heightened sensitivity to social difficulties and external stress due to adverse macroeconomic situations.

**Conclusion:**

Mental health significantly influences college students’ job decisions, with variations noted by gender and enrollment year. External stress and severe psychopathology are crucial factors that either facilitate adaptive coping or impede self-efficacy in the setting of economic adversity. The impacts are most evident in vocational universities, where students’ employment preparation and employment expectations amplify psychological consequences. University administrators should implement mental health interventions and career counseling workshops customized for each gender and enrollment cohort, in order to improve students’ self-efficacy, emotional resilience, and adaptability to fluctuations in the employment market.

## 1 Introduction

Understanding the impact of mental health on career choices is crucial for assisting college students in securing employment. Social Cognitive Career Theory (SCCT) (Lent et al., 1994) indicate that mental health status can influence occupational decisions by altering an individual’s self-efficacy, career aspirations, and goal-setting processes. Approximately one-third of college students globally suffer from mental health issues such as anxiety and depression (Li et al., 2022), and these issues arise from factors such as familial discord, financial difficulties, academic pressure, inability to acclimate to new circumstances (Campbell et al., 2022; Siraji et al., 2022). The Covid-19 pandemic has exacerbated college students’ mental health by necessitating online learning, inducing feelings of isolation, and instilling concerns over future employment (Chen and Lucock, 2022; Li et al., 2024). During the pandemic 60.8% of American college students (Lee et al., 2021) and 40% of Chinese college students (Fu et al., 2021) had signs of anxiety.

## 2 Literature review

The relationship between mental health and career has been well examined. Mental health issues, e.g., anxiety and depression, might diminish occupational self-efficacy, heighten vocational indecision, and restrict an individual’s exploration of many occupational fields (Walker and Peterson, 2012). Conversely, possessing mental health can facilitate career planning and adaptability amidst uncertainties in the labor market (Fang and Xu, 2025; Gao et al., 2025). Mental health issues among college students are intricately linked to occupational anxiety and challenges in decision-making. Anxiety and depression can diminish college students’ perception of professional autonomy and preparedness for career decision-making (Anghel and Gati, 2021; Arbona et al., 2021). Psychological distress indirectly influences college students’ career decisions by diminishing self-efficacy and amplifying employment pressure (Shao et al., 2022). Conversely, students with positive psychological disposition exhibit positive intentions for proactive job-hunting, career goal achievement, and entrepreneurial endeavors (Haddoud et al., 2024; Kwak et al., 2025).

The intersection of digitalization and the COVID-19 epidemic have revealed new characteristics in the manifestations and coping strategies of mental health issues among college students. Digital intervention techniques, like AI chatbots, have demonstrated beneficial outcomes in mitigating psychological issues such as anxiety and depression, offering more accessible and cost-effective psychological care for college students (Lee et al., 2024). Simultaneously, online communities have emerged as a significant platform for youth to articulate their thoughts and alleviate psychological distress (Tuan et al., 2024). College students often employ terminology associated with anxiety and despair, indicating their necessity for social support when confronting psychological stressors like job searching. The COVID-19 pandemic, among others, has intensified psychological problems such as anxiety and loneliness in college students, adversely impacting their perceptions of online learning and intentions regarding career behavior, thereby exacerbating career anxiety and complicating employment decision-making (Abbas et al., 2024).

The influence of mental health on career selection exhibits heterogeneity. Social anxiety and interpersonal sensitivity may lead students to eschew service-oriented or socially demanding professions (Clarke and Fox, 2017; Himle et al., 2020). Impulse and external stress may drive individuals to select self-employment or unconventional occupations (Kottwitz et al., 2025; Wiklund et al., 2018). Men and women are also influenced differently by their mental health conditions when making career decision. Men’s career choices are more influenced by external stress and self-assertion. Conversely, women are more impacted by mental stress and interpersonal issues (Rivera-Torres et al., 2013; Vial and Cowgill, 2022).

Similar to the association between mental health and academic success (Monzonís-Carda et al., 2024), mental health and employment have a bidirectional relationship. Employment serves as an effective means to enhance mental wellbeing, not just in alleviating depression and anxiety but also in improving the conditions of individuals with severe mental illnesses (Modini et al., 2016). Supported employment effectively aids those with severe mental illnesses by fostering independence, enhancing self-esteem, and garnering respect from others (Drake and Wallach, 2020).

Although many research has explored the association between mental health and career development, empirical evidence concerning the impact of certain mental health aspects on distinct job choices is scarce, especially in the context of vocational education. Students at higher vocational universities face more work pressure, lower social status perception, and more utilitarian training environments compared to comprehensive and research-oriented universities (Liu, 2025). In addition, vocational college students also show significant differences in academic motivation, professional identity and psychological adaptation patterns. The enrollment motivation of vocational college students is more utilitarian and they are more employment-oriented. However, there is often a gap between career expectations and actual opportunities, which can easily lead to anxiety, self-doubt and employment pressure (Zhao, 2023). Perceptions of discrimination arising from social bias and occupational hierarchy might undermine the occupational self-efficacy of vocational college students, as indicated by a decrease in occupational exploration and a loss in psychological flexibility (Liu et al., 2022). These characteristics indicate that the mental health status of students at vocational universities often has a more immediate impact on their job-seeking aspirations and career preferences.

This research investigates the influence of mental health on the career choices of college students. This investigation examines many psychological characteristics, including anxiety, depression, and external stress, to elucidate how these factors influence college students’ career choices via mechanisms such as self-efficacy and goal setting. Especially in the context of digitalization and the pandemic, the changes in the psychological state of college students and the particularity of vocational university students in terms of their psychological conditions and employment environment. The aim is to provide targeted mental health intervention and career guidance basis for educational administrators and university counseling systems, and deepen the understanding of the relationship between mental health and career choice.

## 3 Data and methods

### 3.1 Data source and evaluation criteria

This paper uses L University in Fujian Province, China, as the research sample. L University is a public higher vocational institution focused on practical training, with an enrollment of roughly 17,000 students. The selection of vocational universities as the research sample is predicated on their employment-oriented training objectives, contrasting with the research- oriented nature of comprehensive or research universities. Furthermore, the educational environment of vocational universities prioritizes the cultivation of students’ practical skills and directly engages with the job market. Thus, it is more suitable for this paper’s research goal, which is to study how mental health affects career choice.

The mental health data is sourced from the psychological survey administered by the student management department to freshmen each September. Although only one-time data during students’ enrollment period was available, existing research (Liu et al., 2023; Seehuus et al., 2025) has demonstrated the mental health status of college students remains relatively stable during their undergraduate years, and mental health data as the baseline collected at the time of enrollment is a strong predictor of subsequent academic and behavioral outcomes (Beiter et al., 2015; Bruffaerts et al., 2018). Therefore, the use of data at the time of enrollment in the study is unlikely to bias the observed associations between mental health and career choice.

The employment data is obtained from the employment management department. After excluding invalid samples from the mental health data and linking the mental health survey with employment data via student IDs, the final valid sample comprised 10,981 individuals, comprising 2,767 students from 2017, 3,203 from 2018, and 5,011 from 2019. Table 1 displays the demographic features of the sample.

**Table 1:** Demographic characteristics of the sample.

Enrollment year	Gender		Both
	Male	Female	
2017	1,572	1,195	2,767
2018	1791	1,412	3,203
2019	2,835	2,176	5,011
Total	6,198	4,783	10,981

The College Students Mental Health Screening Scale (Fang et al., 2018) is utilized for mental health evaluation. The instrument comprises 96 items encompassing 22 domains, including hallucinations and delusions, suicidal ideation and behaviors, anxiety, depression, paranoia, inferiority, sensitivity, social phobia, somatization, dependence, hostility and aggression, impulsivity, compulsion, internet addiction, self-harm, eating disorders, sleep disturbances, academic adjustment issues, interpersonal relationship distress, academic stress, employment stress, and romantic distress. The scale exhibits an internal consistency reliability (Cronbach’s *α* coefficient) exceeding 0.7 and a test–retest reliability surpassing 0.6. A 4-point Likert scale is employed to evaluate each item, where 1 signifies “not at all like me” and 4 denotes “very much like me.” The overall score for each dimension is obtained by summing the scores of all its elements. The overall score is subsequently converted to a z-score. A higher score indicates a greater severity of psychological issues. Table 2 illustrates the distribution of z-scores for mental health within the sample.

**Table 2:** Mental health variables of the sample.

Variable	z-score				
	Mean	Median	SD	Min	Max
Anxiety	0.50	0.11	1.15	−0.91	4.76
Compulsiveness	0.47	0.35	1.09	−1.05	4.23
Dependence	0.55	0.43	1.08	−0.98	4.43
Depression	0.49	0.21	1.11	−1.02	4.78
Eating disorders	0.10	−0.23	0.95	−0.91	6.47
Hallucinations and delusions	0.29	0.08	1.14	−0.58	7.18
Hostility and aggression	0.20	−0.18	1.06	−0.80	5.89
Impulsivity	0.48	0.65	1.02	−1.11	3.99
Inferiority	0.46	0.22	1.13	−0.96	4.62
Employment stress	0.48	0.65	0.94	−1.28	2.59
Romantic distress	0.19	0.00	1.01	−1.04	4.71
Internet addiction	0.74	0.69	1.12	−1.08	3.97
Paranoia	0.35	0.08	1.07	−0.93	4.82
School adjustment difficulties	0.29	0.18	0.98	−1.15	4.18
Self-harming behavior	0.04	−0.53	0.97	−0.55	7.80
Sensitiveness	0.52	0.64	1.07	−1.16	3.98
Sleep disorders	0.46	0.32	1.09	−1.06	4.15
Social phobia	0.50	0.54	1.19	−0.90	4.73
Interpersonal relationship distress	0.36	0.47	1.02	−1.04	4.69
Somatization	0.18	−0.14	1.09	−0.69	5.65
Academic stress	0.61	0.60	0.95	−1.28	3.23
Suicidal ideation	0.16	−0.40	1.19	−0.41	7.60

SD refers to standard deviation.

Employment sectors are categorized into 19 classifications as per the Chinese national standard GB/T 4754–2017 (Nation Bureau of Statistics, 2017). This article consolidates 19 industries into five groups based on functional similarity, and Labor Market Segmentation Theory (Doeringer and Piore, 1971).

*Agriculture and resource industries (AR),* encompassing agriculture, forestry, animal husbandry, fishery, water resource public facilities management, are sectors grouped due to their reliance on natural resources and primary production activities.

*Finance and public services (FP)* include the finance industry, public administration-social security and social organizations, as well as health and social work, which share similarities regarding employment form and regulatory framework. These careers provide relative stable jobs predicated on qualifications and are frequently associated with government, fiscal system, and the provision of welfare services. The integration of these sectors is grounded in the dual labor market model (Doeringer and Piore, 1971), which categorizes finance and public services as components of the “core” or “institutional” segment of the economy, characterized by high formalization and policy integration.

*Industrial and infrastructural sectors (II)* include industries such as manufacturing, construction, power generation-heating-gas-water production and distribution, and mining. These sectors are grouped due to their emphasis on manufacturing, extensive reliance on technology, and high requirement for engineering and technical labor.

*Information and technology industry (IT)*, encompassing information transmission software, IT services, and scientific research and technical services, is defined by depending on technology and innovation, emphasizing knowledge capital and digital infrastructure. They represent the emerging skill-oriented segment of China’s evolving employment landscape.

*Service industry (SI)* encompasses eight sectors: education, real estate, residential repair services, accommodation and catering, wholesale and retail, leasing and business services, culture-sports and entertainment, and transportation-storage-postal services, which represent the tertiary service economy with flexible employment, customer interaction, and variable income structures.

Additionally, this paper three groups of employment-related status are also classified:

*Unemployment* contains “waiting for employment,” “planning to take civil service exams,” “planning to start a business,” “job hunting,” “in the process of signing a contract,” and “not currently employed.”

*Self-employment (SE)* covers self-employment and freelancing, which represents independent professional endeavors.

*Non-employment (NE)*, including “continuing education,” “studying abroad,” and “mandatory military service.” Even though “continuing education” and “studying abroad” are often viewed as proactive investments in students’ careers, they can still be regarded as career decision-making made to delay entry into the job market. And these two choices are also influenced by psychological and practical factors, e.g., anxiety, self-efficacy, and risk aversion (Shkoler and Rabenu, 2023; Zhou et al., 2024). In other words, such classification is for the sake of analytical comparability and model consistency, rather than as a value judgment.

While these three groups may not constitute conventional employment, they may be shaped by mental health conditions as a decision encountered by graduates with job challenges. Finally, adding the previous five groups, we establish a total of eight industry categories by amalgamating the previously described five industry categories. The allocation of employment sectors within the sample is presented in Table 3.

**Table 3:** Distribution of employment industry in the sample.

Employment industry	Enrollment year			Total
	2017	2018	2019	
Agriculture and resource industries	14	12	39	65
Finance and public services	98	54	86	238
Industrial and infrastructure industries	659	788	916	2,363
Information and technology industry	331	246	352	929
Non-employment	738	970	1906	3,614
Self-employment	29	21	48	98
Service industry	861	1,094	1,595	3,550
Unemployment	37	18	69	124

### 3.2 Method

**Figure 1:**
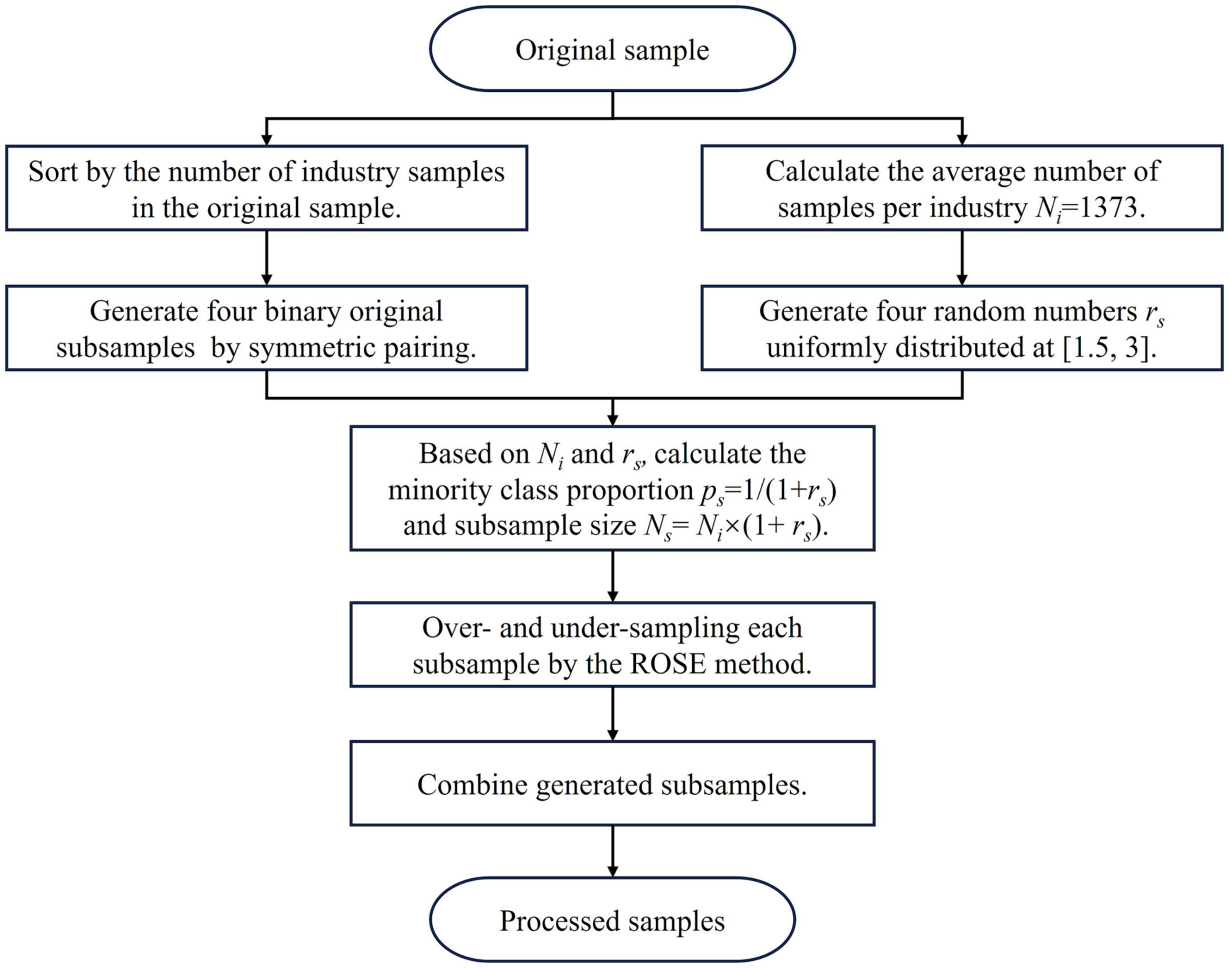
The process of sample over- and under-sampling.

Table 3 illustrates a significant class imbalance in the employment sectors of the sampled students, with the number of samples in the industrial and infrastructure sectors, non-employment, and service industry substantially surpassing those in other sectors. The ROSE approach (Lunardon et al., 2014) by ovun.sample() function in the R package ROSE was employed to execute over-sampling of the minority class and under-sampling of the majority class, thereby ensuring robust results. Given that the ROSE method is exclusively applicable to binary classification samples, the original samples are segmented into four binary classification subsamples via the symmetric pairing technique. Namely, rank each industry in Table 3 in increasing order based on the student count. The first rank (Agriculture and resource industries) and last rank (Non-employment) constitute the first subsample, the second rank (Self-employment) and second to last rank (Service industry) comprise the second subsample, and this pattern continues. In each subsample, the minority class is augmented to match the average sample size of each category in the original samples, while the majority class is maintained at 1.5 ~ 3 times^1^ the size of the minority class. The precise processing sequence is illustrated in Figure 1.

**Figure 2:**
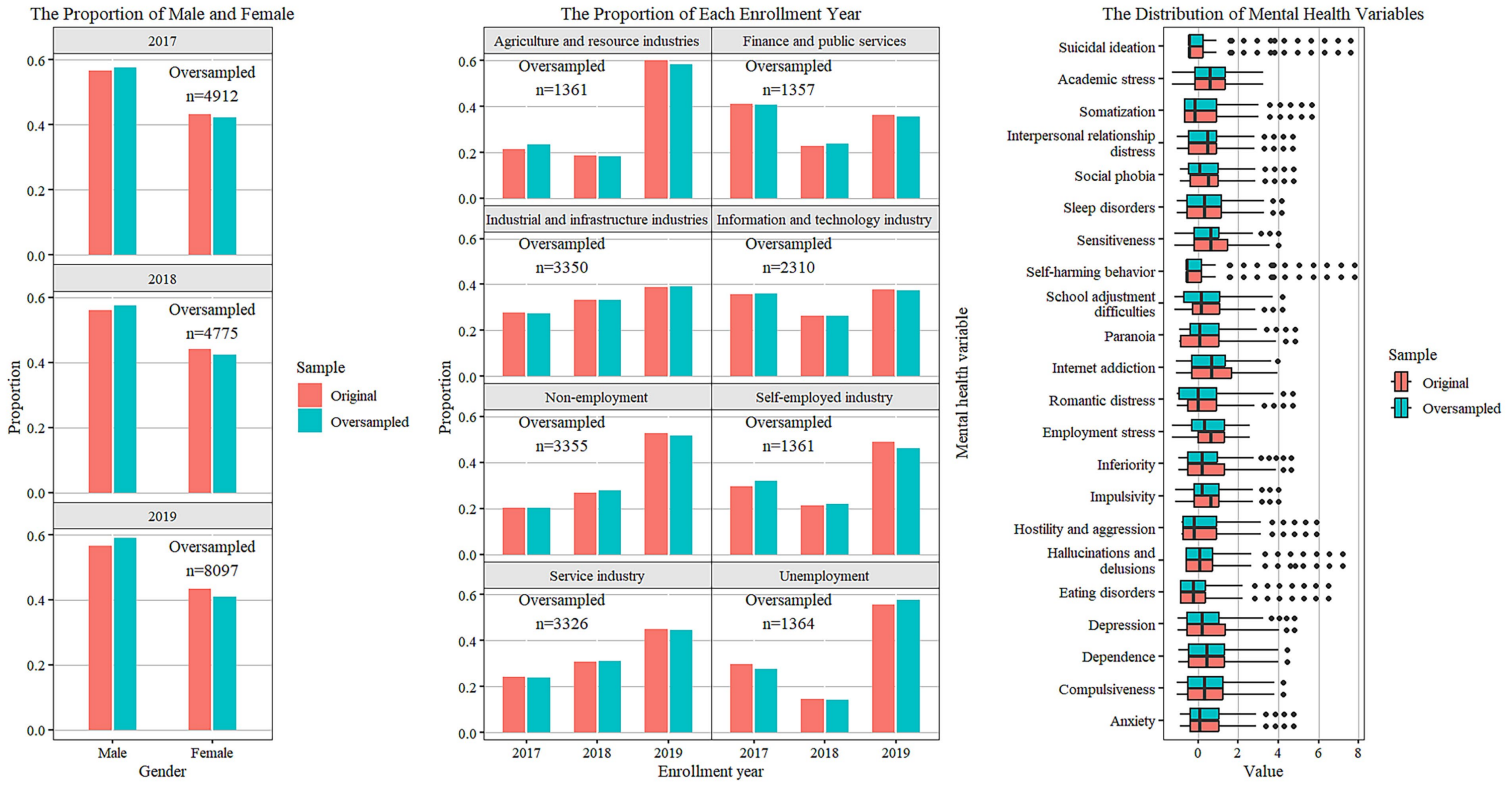
Gender structure and industry distribution in original and oversampled samples.

To ensure that the ROSE method does not distort the association between mental health and career choice in the original samples, we conducted two layers of verification. First, we compared the marginal distributions of gender, enrollment year, industry of employment, and mental health indicators in the samples before and after oversampling in Figure 2, which shows that the distribution of all variables in the synthetic sample align with those of the original sample. Second, we computed the correlation matrices for original and synthetic data, assessing their similarity using Pearson’s matrix correlation coefficient and the Frobenius norm. The results, with Pearson’s matrix correlation coefficient at 0.85 and Frobenius norm at 0.204, indicate that the synthetic sample generated by the ROSE method is structurally congruent with the original sample and preserves the association between mental health and career choices.

We also utilize precision, recall, F1-score, and AUC (area under the curve) to evaluate the model’s performance. We employ stratified sampling to divide the oversampled data into 80% for training and 20% for testing. This procedure ensures that the industries in both the training and test sets are distributed uniformly. We train the Random Forest model using the training set data and then make predictions on the test set data. We evaluate the aforementioned four metrics using the confusion matrix. The precision is 0.833, and the AUC is 0.933, indicating that the classification is highly effective. Table 4 indicates that the model performs effectively across all domains, with macro values exceeding 0.88 and weighted values surpassing 0.83. The three metrics for non-employment and the service industry are diminished; nonetheless, the oversampled data collectively remain viable for further research.

**Table 4:** The precision, recall and F1-score metrics for each industry in oversampled sample.

Career industry	Index		
	Precision	Recall	F1-score
Agriculture and resource industries	1	0.993	0.996
Finance and public services	1	0.971	0.985
Industrial and infrastructure industries	0.766	0.748	0.757
Information and technology industry	0.983	0.866	0.921
Non-employment	0.710	0.711	0.710
Self-employed industry	0.989	0.996	0.993
Service industry	0.705	0.763	0.733
Unemployment	0.922	1	0.960
Macro	0.884	0.881	0.882
Weighted	0.837	0.833	0.834

**Figure 3:**
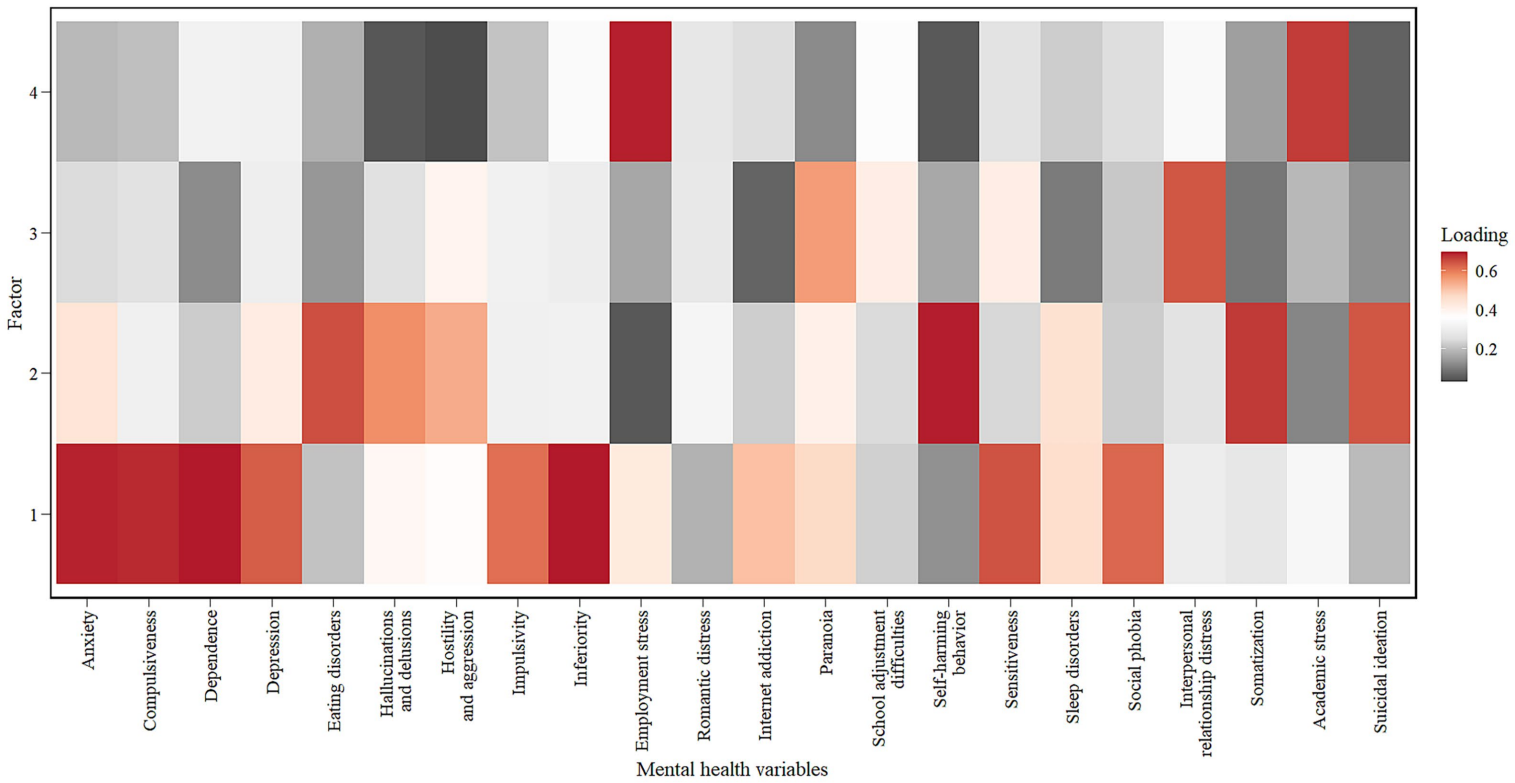
Factor loadings on mental health variables.

The study employs multinomial logistic regression to examine the influence of mental health characteristics on employment decisions and utilizes the likelihood ratio test to assess the significance of the model. The dependent variable comprises the eight industry sectors enumerated in Section 3.1, with unemployment serving as the reference category. The control variables are gender (with female as the reference category) and year of enrollment (with 2017 as the reference year). There are 22 mental health variables, with 190 pairs exhibiting a correlation coefficient exceeding 0.5 (for instance, sensitiveness and paranoia *ρ* = 0.66). Additionally, there exist 36 pairs with a correlation coefficient exceeding 0.7 (for instance, anxiety and compulsiveness *ρ* = 0.73). The variance inflation factor (VIF) for all pairs is below 0.5, which means that even though the 22 mental health factors are correlated, they do not have serious multicollinearity issues, but having so many variables might still lead to some repeated information. The KMO values for all 22 mental health variables are above 0.9, and the results from the Bartlett test are significant, showing that factor analysis is appropriate for finding potential mental health factors that could reduce overlap and improve the model’s performance. We are using the maximum likelihood with orthogonal rotation to extract the components. The parallel analysis suggests the seven components, with the initial four factors accounting for 57% of the variation, satisfying the criterion of explaining at least 50% of the variance (Streiner, 1994). Figure 3 displays the factor loadings.

Factor analysis identified four primary factors:

Factor 1: This factor demonstrates elevated loadings (>0.4) on anxiety, compulsion, dependence, depression, impulsivity, inferiority, internet addiction, sensitivity, sleep disorder, and social phobia. These items primarily reflect internal emotional and cognitive symptoms, leading to the designation of this factor as internalizing symptoms (IS).

Factor 2: Elevated loadings (>0.5) are observed for eating disorders, hallucinations and delusions, hostility and aggression, self-harming behaviors, somatization, and suicidal ideation. This indicates the presence of more severe mental health conditions, which can be classified as severe psychopathology (SP).

Factor 3: This factor demonstrates a substantial loading (>0.4) associated with paranoia, school adjustment difficulties, and Interpersonal relationship distress. These indicators reflect social difficulties and paranoid tendencies; thus, it may be termed social difficulties (SD).

Factor 4: The prominent loadings (>0.6) relate to employment and academic stress, suggesting psychological symptoms stemming from external pressures. We designate this factor as external stress (ES).

Romantic distress (Rmd) reveals low loadings across all four categories (<0.4) and is categorized as an emotional element, based on theoretical rather than solely statistical criteria. The SCCT posits that as an emotional input, as an emotional input, emotional states such as romantic distress can directly or indirectly affect an individual’s self-efficacy and outcome expectations, thereby shaping an individual’s occupation-related behaviors (Lent et al., 2017). Romantic distress is a unique type pf interpersonal emotional tension that cannot be represented by general internalized symptoms or social difficulties. Romantic relationship stress can, on one hand, disrupt the hierarchy and priority of goals across various life domains, thereby affecting the clarity and stability of career decisions (Ranta et al., 2014). On the other hand, it can reduce an individual’s focus and execution ability regarding career objectives through the transactive goal dynamics mechanism, exacerbating psychological burdens and goal conflicts (Kornblum et al., 2021). Therefore, although loadings on Romantic distress are low, it represents the emotional stress an individual experiences and can still be regards as a valuable variable, which influencing students’ career decision-making process.

We constructed an analytical framework based on the SCCT, which highlights how self-efficacy, outcome expectations, and contextual factors interact to influence career choices. Although the samples used in this article do not include indicators that directly measure self-efficacy or outcome expectations, the above-mentioned mental health factors can serve as operational indicators for both: *social difficulties* and *external stress*, which represent perceived situational barriers that may impair self-efficacy; *internalized symptoms* (e.g., anxiety and depression) represent emotional states that may undermine confidence or an optimistic attitude toward career; *romantic distress* indicate interpersonal relationship pressure, which can affect goal persistence and perceived attainability of future outcomes.

Based on this, this paper proposes three models: Model 1 (benchmark model) includes two control variables: gender and year of enrollment. This model aims to assess how gender and background factors influence the job decisions of college students. Model 2 builds upon Model 1 by incorporating mental health variables (IS, SP, SD, ES, and Rmd) to investigate the effects of mental health characteristics on job decisions. Model 3 further develops Model 2 by adding interaction terms for gender, year of enrollment, and mental health factors to explore how these interactions affect employment decisions.

## 4 Results

### 4.1 Model 1

Table 5 presents the outcomes of Model 1 (McFadden *R*^2^ = 0.026, AIC = 69156.2). The likelihood ratio test results [*χ*^2^(21) = 1837.5, *p* < 0.001] indicate that the model is statistically significant. The evidence indicates that gender and enrollment year significantly influence job choices in comparison to unemployment status. Male exhibit a lower propensity than female to select agriculture and resource industries (OR = *e*^-0.613^ = 0.542), finance and public services (OR = 0.217), non-employment (OR = 0.609), and service industry (OR = 0.328), although they demonstrate a higher likelihood of opting for self-employment (OR = 1.372). Individuals do not significantly select careers in the industrial and infrastructure sectors or the information and technology industry.

**Table 5:** The impact of gender, enrollment year on employment choice.

Variable	AR	FP	II	IT	NE	SE	SI
Intercept	0.208* (0.091)	1.147*** (0.081)	0.922*** (0.077)	0.839*** (0.080)	0.894*** (0.078)	−0.087 (0.094)	1.365*** (0.076)
Male	−0.613*** (0.080)	−1.530*** (0.083)	−0.055 (0.070)	−0.083 (0.074)	−0.496*** (0.069)	0.316*** (0.086)	−1.113*** (0.069)
EY2018	0.410*** (0.122)	0.122 (0.114)	0.857*** (0.099)	0.357*** (0.103)	0.984*** (0.101)	0.277* (0.116)	0.927*** (0.100)
EY2019	0.181* (0.091)	−0.845*** (0.089)	−0.375*** (0.076)	−0.686*** (0.079)	0.216** (0.077)	−0.373*** (0.088)	−0.101 (0.077)

Standard errors in parentheses, ***p < 0.001 Extremely significant, **p < 0.01 Highly significant, *p < 0.05 Significant. The same below.

In terms of enrollment year, students who enrolled in 2018 showed a greater tendency to choose agriculture and resource industries (OR = 1.507), industrial and infrastructure industries (OR = 2.356), information and technology industry (OR = 1.429), non-employment (OR = 2.675), self-employment (OR = 1.319), and service industry (OR = 2.526). However, there was no significant difference in the selection of finance and public services. Students who enrolled in 2019 demonstrated a higher likelihood of selecting non-employment (OR = 1.241) and agriculture and resource industries (OR = 1.198), while showing a decreased likelihood of choosing finance and public services (OR = 0.430), industrial and infrastructure industries (OR = 0.687), information and technology industry (OR = 0.504), and self-employment (OR = 0.689). The selection of service industries exhibited minimal variation.

### 4.2 Model 2

Model 2 (McFadden *R*^2^ = 0.030, AIC = 68944.3) incorporates five mental health factors in addition to Model 1. The likelihood ratio test indicates that the model is significant [*χ*^2^(56) = 2156.3, *p* < 0.001]. Likelihood ratio (LR) tests between Model 2 and Model 1 results in Δ*χ*^2^(28) = 267.9, *p* < 0.001, as well as a larger McFadden R^2^ and a smaller AIC than Model 1, which demonstrates that adding mental health variables improves the model fit performance. Table 6 indicates that the influences of gender and year of enrollment are mostly consistent with those observed in Model 1. Nonetheless, several coefficients have altered. The coefficient for males in the agriculture and resource industries decreased from −0.613 to −0.472. The decrease indicates that psychological health issues partially account for the disparities between male and female.

**Table 6:** The impact of gender, enrollment year and mental health status on employment choice.

Variable	AR	FP	II	IT	NE	SE	SI
Intercept	0.096 (0.092)	1.070*** (0.082)	0.849*** (0.078)	0.768*** (0.081)	0.834*** (0.079)	−0.074 (0.094)	1.293*** (0.077)
Male	−0.472*** (0.084)	−1.464*** (0.086)	0.080 (0.073)	0.064 (0.077)	−0.419*** (0.071)	0.317*** (0.089)	−1.016*** (0.071)
EY2018	0.463*** (0.123)	0.162 (0.114)	0.889*** (0.099)	0.388*** (0.103)	1.015*** (0.102)	0.267* (0.116)	0.960*** (0.101)
EY2019	0.221* (0.092)	−0.807*** (0.090)	−0.364*** (0.077)	−0.680*** (0.080)	0.242** (0.078)	−0.391*** (0.089)	−0.080 (0.078)
Rmd	−0.045 (0.048)	0.139** (0.048)	−0.015 (0.041)	−0.066 (0.044)	0.075 (0.041)	−0.078 (0.050)	0.079 (0.041)
IS	0.047 (0.045)	0.044 (0.046)	0.039 (0.039)	0.059 (0.041)	−0.001 (0.039)	−0.033 (0.047)	0.087* (0.039)
SP	−0.207*** (0.050)	−0.135** (0.049)	−0.032 (0.040)	−0.011 (0.042)	−0.101* (0.040)	0.016 (0.048)	−0.048 (0.040)
SD	0.191*** (0.051)	−0.007 (0.053)	−0.099* (0.044)	−0.074 (0.047)	−0.016 (0.044)	−0.107* (0.054)	−0.138** (0.045)
ES	0.305*** (0.054)	0.167** (0.054)	0.335*** (0.045)	0.350*** (0.048)	0.205*** (0.045)	0.026 (0.053)	0.226*** (0.045)

Standard errors in parentheses, ***p < 0.001 Extremely significant, **p < 0.01 Highly significant, *p < 0.05 Significant.

The influence of mental health factors differs between sectors. *Romantic distress* markedly elevates the probability of selecting finance and public services (OR = 1.149), perhaps due to emotional influences on the self-efficacy associated with employment in public services (Lent et al., 1994). *Internalizing symptoms* exerts a marginal favorable effect on the selection of the service industry (OR = 1.091), with no significant impact on other sectors, suggesting that the influence of this mental health factor is constrained. *Severe psychopathology* markedly diminishes the probability of selecting agriculture and resource industries (OR = 0.813), finance and public services (OR = 0.874), and non-employment (OR = 0.904). *Social difficulties* exert a considerable favorable influence on the selection of agriculture and resource industries (OR = 1.210), while negatively affecting industrial and infrastructure sectors (OR = 0.906), self-employment (OR = 0.899), and service industry (OR = 0.871). The evidence suggests that social difficulties may impede students from accessing industries that necessitate advanced social skills. *External stress* markedly elevates the likelihood of selecting all industries except self-employment, suggesting that external pressure may, to a certain degree, encourage varied career aspirations (Lent et al., 1994).

### 4.3 Model 3

Model 3 (McFadden *R*^2^ = 0.042, AIC = 68267.5) incorporates the interaction terms of mental health factors with gender and enrollment year from Model 2 and the results are listed in Table 7. The likelihood ratio test yielded *χ*^2^(161) = 3072.7, *p* < 0.001, indicating the model’s significance. Likelihood ratio (LR) tests between Model 3 and Model 2 results in Δ*χ*^2^(84) = 844.8, *p* < 0.001, with the largest McFadden R^2^ and the smallest AIC among three models, which indicates that the inclusion of interaction terms enhanced the model’s explanatory power and elucidated significant variance in employment choices beyond demographic and main-effect frameworks.

**Table 7:** The impact of gender, enrollment year, mental health status and their interactions on employment choice.

Variable	AR	FP	II	IT	NE	SE	SI
Intercept	−0.047 (0.106)	1.180*** (0.088)	0.996*** (0.084)	0.876*** (0.087)	0.946*** (0.085)	0.042 (0.100)	1.429*** (0.082)
Male	−0.450*** (0.096)	−1.520*** (0.094)	−0.053 (0.079)	−0.029 (0.084)	−0.509*** (0.079)	0.226* (0.097)	−1.131*** (0.079)
EY2018	0.649*** (0.143)	0.419** (0.129)	1.164*** (0.114)	0.644*** (0.118)	1.298*** (0.117)	0.409** (0.136)	1.212*** (0.116)
EY2019	0.326** (0.100)	−0.742*** (0.094)	−0.256** (0.080)	−0.578*** (0.083)	0.343*** (0.082)	−0.397*** (0.095)	0.005 (0.081)
Rmd	0.155 (0.116)	0.337*** (0.101)	0.206* (0.098)	0.142 (0.100)	0.296** (0.098)	0.069 (0.119)	0.216* (0.095)
IS	−0.031 (0.107)	0.147 (0.096)	0.048 (0.092)	0.042 (0.095)	0.062 (0.093)	−0.194 (0.113)	0.247** (0.090)
SP	−0.851*** (0.139)	−0.349*** (0.105)	−0.200* (0.099)	−0.243* (0.103)	−0.307** (0.101)	−0.169 (0.119)	−0.201* (0.097)
SD	−0.294* (0.128)	0.003 (0.109)	−0.233* (0.105)	−0.261* (0.108)	0.063 (0.105)	0.333** (0.125)	−0.110 (0.102)
ES	−0.119 (0.123)	−0.624*** (0.109)	−0.560*** (0.104)	−0.229* (0.107)	−0.592*** (0.105)	−0.471*** (0.125)	−0.618*** (0.102)
Male∙ Rmd	0.417*** (0.099)	0.088 (0.101)	0.230** (0.085)	0.196* (0.090)	0.163 (0.083)	0.312** (0.110)	0.290*** (0.083)
Male∙ IS	0.266** (0.094)	0.142 (0.097)	−0.031 (0.083)	−0.021 (0.087)	0.033 (0.081)	0.320** (0.106)	−0.192* (0.081)
Male∙ SP	0.661*** (0.105)	0.361*** (0.103)	0.296*** (0.083)	0.340*** (0.089)	0.362*** (0.082)	0.520*** (0.110)	0.298*** (0.082)
Male∙ SD	−0.279** (0.107)	−0.424*** (0.112)	−0.262** (0.094)	−0.110 (0.099)	−0.279** (0.092)	−0.637*** (0.118)	−0.236* (0.092)
Male∙ ES	−0.204 (0.115)	0.715*** (0.116)	0.898*** (0.098)	0.628*** (0.103)	0.716*** (0.096)	0.564*** (0.121)	0.823*** (0.096)
EY2018∙ Rmd	−0.291 (0.150)	−0.283* (0.140)	−0.416*** (0.124)	−0.320* (0.130)	−0.358** (0.127)	−0.259 (0.148)	−0.314* (0.125)
EY2019∙ Rmd	−0.647*** (0.117)	−0.456*** (0.113)	−0.537*** (0.099)	−0.519*** (0.104)	−0.496*** (0.100)	−0.616*** (0.118)	−0.479*** (0.099)
EY2018∙ IS	−0.106 (0.157)	0.310* (0.145)	0.451*** (0.128)	0.592*** (0.133)	0.489*** (0.131)	0.401** (0.150)	0.479*** (0.130)
EY2019∙ IS	−0.103 (0.107)	−0.363*** (0.106)	−0.037 (0.091)	−0.072 (0.095)	−0.261** (0.093)	−0.221* (0.107)	−0.280** (0.092)
EY2018∙ SP	0.309 (0.161)	−0.301* (0.139)	−0.218 (0.118)	−0.270* (0.125)	−0.283* (0.123)	−1.046*** (0.163)	−0.303* (0.119)
EY2019∙ SP	0.266 (0.141)	0.138 (0.120)	0.037 (0.104)	0.142 (0.107)	0.078 (0.106)	0.044 (0.117)	0.057 (0.103)
EY2018∙ SD	0.197 (0.166)	−0.425** (0.153)	−0.056 (0.131)	−0.185 (0.137)	−0.395** (0.134)	−0.381* (0.157)	−0.257 (0.133)
EY2019∙ SD	0.996*** (0.128)	0.526*** (0.120)	0.489*** (0.104)	0.479*** (0.108)	0.260* (0.105)	−0.039 (0.122)	0.216* (0.105)
EY2018∙ ES	0.507** (0.156)	0.278 (0.150)	0.183 (0.130)	−0.067 (0.135)	0.229 (0.133)	−0.030 (0.154)	0.252 (0.132)
EY2019∙ ES	0.643*** (0.117)	0.558*** (0.120)	0.449*** (0.101)	0.285** (0.105)	0.491*** (0.102)	0.167 (0.118)	0.500*** (0.102)

Standard errors in parentheses, ***p < 0.001 Extremely significant, **p < 0.01 Highly significant, *p < 0.05 Significant.

The male coefficient in self-employment in Model 3 was less significant than in Model 2 (from *p* < 0.001 to *p* < 0.05), suggesting that the interaction term partially altered certain gender effects. The significance of some industrial coefficients varied the years of enrollment. For the 2018 cohort, the coefficients of finance and public services changed from non-significant to highly significant, while self-employment advanced from significant to very significant. For the 2019 cohort, the coefficients of agriculture and resource industries and non-employment attained high and extreme significance, respectively. These findings suggest that the interaction terms enhanced the interpretation of e temporal and gender-specific variations.

The interaction term significantly altered the primary effect of the mental health factors. *Romantic distress* become—significant positive influence on finance and public services in Model 2—positively associated the industrial and infrastructure sectors (OR = 1.229), non-employment (OR = 1.344), and service industry (OR = 1.241). This reflects that emotional distress from romantic experiences may amplify individuals’ motivation to pursue stable or emotionally fulfilling career paths. *Internalizing symptoms* strengthened the association with service industry, indicating that individuals with anxiety or depressive tendencies may favor relational or emotionally expressive work settings. *Severe psychopathology* negatively affects all occupations except self-employment, indicating that serious mental health disorders limits employment opportunities despite contextual moderations. *Social difficulties* diminished the likelihood of choosing agriculture and resource, industrial and infrastructure, and the information and technology industries (OR = 0.745 ~ 0.792) but increased the probability of self-employment (OR = 1.395), implying that interpersonal barriers may compel certain students, especially males, toward self-employed work. The beneficial effect of *external stress* in Model 2 weakened in Model 3, significantly lowering the likelihood of selecting careers besides agriculture and resource industries, which may be attributed to gender-specific variations in stress perception and coping mechanisms (Matud, 2004).

The gender interaction term significantly moderated the effects of mental health factors on career choices. For males, *romantic distress* markedly elevates the likelihood of selecting agriculture and resource (OR = 1.517), industrial and infrastructure (OR = 1.259), information and technology (OR = 1.217), service industries (OR = 1.336), and self-employment (OR = 1.366). The pattern suggests that emotional distress may drive male students to hunt jobs with autonomy or practical engagement, aligning with gender role expectations that prioritize instrumental coping and goal-oriented behavior. *Internalizing symptoms* encourage men to select agriculture and resource industries (OR = 1.305) and self-employment (OR = 1.377), while inhibiting them from choosing service industry (OR = 0.825). On the contrary, *severe psychopathology* exerts a less pronounced restricting influence on men compared to women, indicating that men’s occupational participation may be less affected by clinical-level symptoms. Moreover, *social difficulties exert* a more adverse impact on men’s selection of the sectors besides agriculture and resource, whereas *external stress* elevates the likelihood of men opting for nearly all careers except for agriculture and resource industries. The aforementioned results suggest that gender socialization and coping strategies shape the influence of mental health issues on employment behavior—men generally adopt externalized or behavior-focused strategies, such as self-employment, whereas women tend to retreat from high-demand work atmosphere.

The interaction with the enrollment year moderated the association between mental health and career choice, reflecting how macroeconomic and contextual adaptation work. *Romantic distress* has less impact on students enrolling in 2018 and 2019 than on those in 2017, reflecting that later cohort may develop greater emotional resilience or possessed more pragmatic expectations regarding the job market. *Internalizing symptoms* significantly increased the likelihood that students enrolling in 2018 selected non-agriculture sectors, possibly reflecting adaptive coping strategies in reaction to early pandemic disturbances. Nonetheless, they diminished the probability that students enrolling in 2019—graduating at the period of economic downturn—chose finance and public services (OR = 0.696), non-employment (OR = 0.770), self-employment (OR = 0.802), and the service sector (OR = 0.756). This implies that depression and anxiety intensified under heightened labor-market pressure. Similarly, *severe psychopathology* prevented students enrolling in 2018 from engaging in all sectors except for agriculture and resource or industrial and infrastructure industries. Its impact was diminished for the 2019 cohort, perhaps due to the scarcity of employment rendering mental health disparities less significant. *Social difficulties* significantly diminished the 2018 cohort’s likelihood of selecting finance and public services (OR = 0.654), non-employment (OR = 0.674), or self-employment (OR = 0.683), but increased the 2019 cohort’s probability of engaging in other sectors, suggesting compensatory adaptation in the face of ongoing uncertainty. *External stress* increased the likelihood of 2018 entrants selecting agriculture and resources (OR = 1.660) and steered 2019 entrants toward more stable occupational categories, reflecting that contextual stress exerted a more significant behavioral influence in adverse economic conditions.

## 5 Discussion

This study uses multinomial logistic regression to examine how college students’ mental health shape their career decisions. The results reflect that gender, year of enrollment, and five psychological factors—romantic distress, internalizing symptoms, severe psychopathology, social difficulties, and external stress—are associated with unique patterns of career choice and interaction effects.

Model 1 (benchmark model) suggests that gender and enrollment year affect career selection. Men appear less inclined to pursue careers in female-dominated sectors, such as finance and public services, and the service industry. They are more likely to pursue self-employment, which is consistent with gender role stereotypes (Eccles, 2009) and occupational gender segregation (Blau and Kahn, 2017). Such results reflects that self-efficacy and outcome expectations—the fundamental ideas of the Social Cognitive Career Theory—play different roles in male and female job decision-making behaviors. Higher self-efficacy in taking risks and a belief in positive outcome expectations linked to autonomy and achievement may prompt male students to have stronger entrepreneurial intentions (Thébaud, 2010), rather than choose female-dominated sectors, which are viewed as underappreciated and underpaid due to their emphasis on emotional labor.

Nonetheless, these gender-based distinctions must be comprehended within the particular cultural and institutional framework of Chinese higher vocational education, where gendered social norms and job arrangements may diverge from those in other countries or educational systems. Vocational education prioritizes practical skills and swift transitions from campus to workplace to expedite the development of career identities; yet, it also results in “internalized disparities between gender and occupation.” For example, male students pursuing engineering and applied technology often internalize societal expectations of autonomy and competitiveness, whereas female students in service-oriented majors may experience increased anxiety or role tension due to the emphasis on emotional labor.

Besides gender, students’ career choices are influenced by macroeconomic contexts. Students enrolled in 2018 had a higher likelihood of employment compared to those enrolled in 2017. The 2018 cohort have graduated in 2021 with GDP growth rate of 8.4 percent (National Bureau of Statistics, 2022), a period characterized by a more favorable economy compared to 2020 when the 2017 cohort graduated and more conducive to employment. However, students who enrolled in 2019 and graduated in 2022 encountered economic instability and heightened rivalry in the employment market. GDP growth was only 3.0%, far below the official target of “around 5.5%” (Yao and Zhang, 2023), the unemployment rate among urban youth aged 16–24 reached a new high of 19.9%, and the number of college graduates surpassed 10.76 million at the same time (Cheng, 2022). Although General Office of State Council (2022) issued a special notice that increased policy support for college graduates’ employment to stabilizing graduate employment, these macroeconomic pressures increased competition and uncertainty—causing the 2019 cohort to make more cautious and risk-averse decisions. This pattern can be explained by SCCT contextual constraints framework that economic downturns can change individuals’ outcome expectations, lower their self-efficacy, and make them feel like there are fewer chances. Importantly, for vocational students whose training is closely linked to labor market demand, macroeconomic downturns are experienced more acutely, intensifying the stress—career decision correlation identified in this study.

Model 2 implies that psychological factors may play different roles in shaping students’ career decision-making behaviors. Internalized symptoms and severe psychopathology, as negative proxies of self-efficacy, undermine students’ confidence in facing job-hunting problems. Social difficulties and romantic distress, representing perceived barriers, may reduce individuals’ optimistic outcome expectations regarding future employment and lead to preferences for specific occupations. These associations imply that self-efficacy and the expected outcomes of career decisions—two essential elements of the SCCT framework—are influenced by emotional and interpersonal factors. Under economic instability and high job rivalry, external stress may enable some individuals to enhance their adaptability and explore a broader array of employment options, aligning with SCCT’s focus on adaptive coping and goal revision that enable individuals to adjust goals in response to environmental constraints.

The aforementioned pattern is significant in the in the vocational education atmosphere. Vocational education focused on application and performance frequently converts external pressures into learning assignments. Students experiencing mental health issues may perceive this pressure as a danger, resulting in avoidance behavior or early disengagement. Consequently, the practice-oriented vocational education framework accentuates the dual nature of external pressure as both a motivator and a hazard.

Model 3 suggests that cohort context and gender could moderate how mental health shape career decisions. Men generally exhibit proactive or compensating work actions in reaction to emotional and stress-related factors, while women appear to be more constrained by internalized distress, which implies gender differences in self-efficacy and coping strategy. Economic and institutional circumstances, reflected in cohort differences, could either exacerbate or alleviate the effects of specific mental health issues. For example, the 2019 cohort, graduating in an adverse period, exhibited increased reactions to external stress and social difficulties, possibly as compensatory coping strategies. This pattern also aligns with SCCT that contextual affordances and barriers dynamically influence career decision-making processes, external stress and social challenges seemed to trigger compensatory coping responses in the cohort which experience intensified job market pressures when graduating.

This compensatory coping response will be reinforced by the “employment preparation” ambiance in the vocational education setting, leading students to associate employability with self-discipline and adaptability. This institutional reinforcement may elucidate why, in this study, external pressure serves as both a motivation and a cause of anxiety: students are trained to utilize more pragmatic tactics in stressful contexts, while they lack adequate emotional support to sustain mental health wellbeing.

Overall, the findings support the Social Cognitive Career Theory, revealing that mental health factors shape career choice directly and indirectly through self-efficacy, perceived barriers, and contextual affordances that interacts with gender and time. Moreover, this influence mechanism is not static but varies according to gender and the socio-economic environment. However, vocational education focused on practical application and employment influences these mechanisms by enhancing the impact of mental health and environmental factors on career choices. The association between stress, coping strategies, and career choices not only illustrates an individual’s psychological processes but also the fundamental principles of vocational training, specifically, employment pressure, task performance, and external evaluation collectively shape students’ mental health and career decisions.

## 6 Conclusion

**Figure 4:**
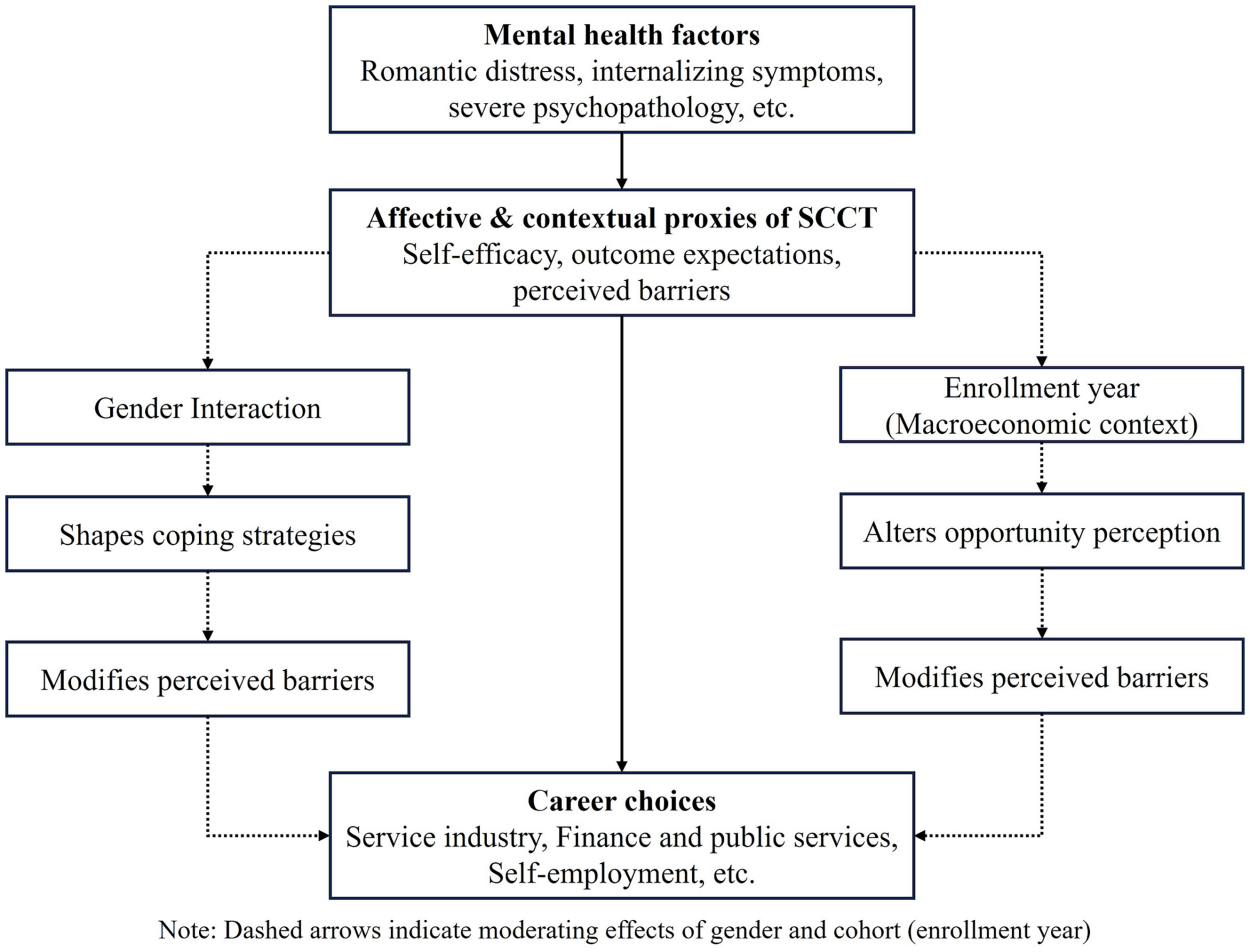
Theoretical framework connecting mental health, gender, and contextual factors to career choices through the SCCT model.

This paper’s findings indicate that mental health shapes college students’ career choices through the interaction of individual psychological states and environmental factors. More precisely, gender and enrollment year moderate the effect of mental health factors, e.g., romantic distress, external stress, and severe psychopathology, on career decisions. The findings underscore that self-efficacy, outcome expectations, and perceived barriers—fundamental elements of the SCCT—mediate how students convert psychological conditions into career behaviors under varying macroeconomic and contextual pressures. Figure 4 displays how mental health factors shapes students’ career choices through the SCCT. Mental health factors function as affective and contextual antecedents that influence self-efficacy, outcome expectations, and perceived barriers. This SCCT construct interacts with macroeconomic contexts that are specific to gender and cohort, which in turn leads to different employment outcomes.

Based on the interaction of mental health factors with gender and environment, we present the following recommendations: Firstly, it is essential to include gender variations in the design of mental health intervention to improve the adaptive coping strategies of both male and female students. Males experiencing external pressures and romantic distress may benefit from career planning workshops by converting emotional stress into proactive goal setting, whereas females suffering from internalizing symptoms may require support in emotional management, social reinforcement, and self-efficacy enhancement.

Secondly, vocational counselors need to use tailored strategies for different cohorts, especially for students who graduates during a time of economic downturn. University administrators should integrate career counseling with stress management training so that students can better understand external uncertainties and view them as manageable tasks rather than insurmountable obstacles.

Thirdly, early vocational education commencing at enrollment should incorporate mental health screening and specialized counseling to avert the decline of mental health, which may impede career exploration.

Finally, the education department and employers must collaborate to foster an inclusive recruitment atmosphere, mitigate the stigma associated with mental health issues, and consequently lower the threshold for the transition from university to workplace.

Furthermore, it is important to consider the distinct features of students from higher vocational institutions when interpreting the results. Students at vocational universities generally face more rigorous practical training requirements, lower perceived social status, and greater employment pressure than their counterparts at comprehensive universities. The impact of mental health issues, e.g., anxiety, social sensitivity, or external stress on career decision-making processes are amplified by these contextual and psychological characteristics. Their pragmatic motivations and greater employment orientation align with SCCT’s contextual emphasis on the interaction between environmental factors and personal agency, making them especially sensitive to changes in opportunity structures and external stress. Therefore, the mechanism obtained in this paper should be regarded as the result of a specific environment rather than a universal causal relationship. Regional differences and variations in the educational environment of universities may further generate an interaction between mental health and environmental factors, influencing student’ employment choices. When applying this finding to other higher education settings or formulating relevant policy recommendations, such background differences must be considered to ensure appropriate extension and avoid excessive generalization.

Even with these boundaries, the findings still provide valuable insights into the influence of mental health on career decisions. Self-efficacy and outcome expectations, as delineated by the Social Cognitive Career Theory (SCCT), along with contextual affordances and several psychological factors, are not exclusive to vocational college students. They may serve as a theoretical framework for comparative and cross-institutional studies, offering significant insights into how the mental health of students under various higher education and socioeconomic environments influences their career decisions. While self-efficacy and outcome expectancies are not explicitly incorporated in the model of this paper, mental health factors serve as surrogate indicators for measuring emotions and situations, hence elucidating the psychological mechanisms of SCCT indirectly. This alignment improves the theoretical coherence of the model and supports the interpretation that emotional and environmental elements jointly affect students’ career decision-making processes.

This study also has some limits. First, mental health data was collected by students’ self-assessments, which may result in social desirability bias and underreporting of severe symptoms. Students may understate socially stigmatized conditions or overstate their positive state to conform to others’ expectations. Students with severe psychopathology may conceal the severity of their symptoms due to embarrassment or privacy concern. These biases may have led to a more conservative evaluation of the relationship between mental health and employment selection, particularly underestimating the impact of severe psychopathology. Future research can enhance measurement validity by combining self-assessment with objective data (e.g., consultation records, behavioral data, and longitudinal surveys) and using social desirability scale to address potential response biases.

Second, while current research indicates that the mental health of college students remains relatively stable throughout their undergraduate years, it is not without variations. This study solely employs the mental health data collected at enrollment and may not reflect the changes in the mental health of college students during their undergraduate experience. Consequently, the estimations derived from static data in this paper may underestimate the dynamic impact of mental health on employment decisions. Subsequent research should amalgamate longitudinal data with mixed-effects models to elucidate the evolution of mental health and their impact on occupational decisions.

Third, family background and economic conditions were not included, and financial strain may exacerbate external stress. Future studies should integrate socioeconomic factors of individuals to further elucidate the contextual mechanisms linking mental health and career decisions.

## Data Availability

The data analyzed in this study is subject to the following licenses/restrictions: the dataset may only be accessed by authorized individuals within the University mentioned in the manuscript. External sharing or distribution is not permitted without prior approval. The data is provided strictly for academic or research purposes and cannot be used for commercial gain. The data contains sensitive personal information that must be anonymized before any analysis or publication. Identifiable information should be removed or encrypted to protect individuals’ privacy. The synthetic data by ROSE method in this paper can be downloaded in supplementary. Requests to access these datasets should be directed to dengx_tutusky@outlook.com.

## Footnotes

1
https://developers.google.com/machine-learning/crash-course/overfitting/imbalanced-datasets

## References

[ref1] Abbas S. Yaseen M. Ameen M. Pervaiz B. Fatima S. Hassan S. (2024). The nexus of Covid-19 and behavioral intentions of university students towards online education. Emerg. Sci. J. 8, 20–34. doi: 10.28991/ESJ-2024-SIED1-02

[ref2] Anghel E. Gati I. (2021). The associations between career decision-making difficulties and negative emotional states. J. Career Dev. 48, 537–551. doi: 10.1177/0894845319884119

[ref3] Arbona C. Fan W. Phang A. Olvera N. Dios M. (2021). Intolerance of uncertainty, anxiety, and career indecision: a mediation model. J. Career Assess. 29, 699–716. doi: 10.1177/10690727211002564

[ref4] Beiter R. Nash R. McCrady M. Rhoades D. Linscomb M. Clarahan M. . (2015). The prevalence and correlates of depression, anxiety, and stress in a sample of college students. J. Affect. Disord. 173, 90–96. doi: 10.1016/j.jad.2014.10.054, PMID: 25462401

[ref5] Blau F. D. Kahn L. M. (2017). The gender wage gap: extent, trends, and explanations. J. Econ. Lit. 55, 789–865. doi: 10.1257/jel.20160995

[ref6] Bruffaerts R. Mortier P. Kiekens G. Auerbach R. P. Cuijpers P. Demyttenaere K. . (2018). Mental health problems in college freshmen: prevalence and academic functioning. J. Affect. Disord. 225, 97–103. doi: 10.1016/j.jad.2017.07.044, PMID: 28802728 PMC5846318

[ref7] Campbell F. Blank L. Cantrell A. Baxter S. Blackmore C. Dixon J. . (2022). Factors that infuence mental health of university and college students in the UK: a systematic review. BMC Public Health 22, 1778–1799. doi: 10.1186/s12889-022-13943-x 36123714 PMC9484851

[ref8] Chen T. Lucock M. (2022). The mental health of university students during the COVID-19 pandemic: An online survey in the UK. PLoS One 17:e0262562. doi: 10.1371/journal.pone.0262562, PMID: 35020758 PMC8754313

[ref9] Cheng S. (2022). China’s youth unemployment rate rises to another record. Caixin. Available online at: https://www.caixinglobal.com/2022-08-15/chinas-youth-unemployment-rate-rises-to-another-record-101926530.html

[ref10] Clarke J. Fox J. (2017). The impact of social anxiety on occupational participation in college life. Occup. Ther. Ment. Health 33, 31–46. doi: 10.1080/0164212X.2016.1222323

[ref11] Doeringer P. B. Piore M. J. (1971). Internal labor markets and manpower analysis: D.C. Heath and Company.

[ref12] Drake R. E. Wallach M. A. (2020). Employment is a critical mental health intervention. Epidemiol. Psychiatr. Sci. 29, 1–3. doi: 10.1017/S2045796020000906, PMID: 33148366 PMC7681163

[ref13] Eccles J. (2009). Who am i and what am i going to do with my life? Personal and collective identities as motivators of action. Educ. Psychol. 44, 78–89. doi: 10.1080/00461520902832368

[ref14] Fang H. Xu X. (2025). The role of psychological resilience and career adaptability in the relationship between perceived social support and employment anxiety among college students. J. Psychol. Afr. 35, 151–157. doi: 10.32604/jpa.2025.065786

[ref15] Fang X. Yuan X. Hu W. Deng L. Lin X. (2018). The development of college students mental health screening scale. Stud. Psychol. Behav 16, 111–118.

[ref16] Fu W. Yan S. Zong Q. Luxford D. A. Song X. Lv Z. . (2021). Mental health of college students during the COVID-19 epidemic in China. J. Affect. Disord. 280, 7–10. doi: 10.1016/j.jad.2020.11.032 PMC7656159 33197782

[ref17] Gao L. Li Y. Pang W. (2025). Career adaptability and graduates’ mental health: the mediating role of occupational future time perspective in higher education in China. BMC Psychol. 13:158. doi: 10.1186/s40359-025-02433-5, PMID: 39988675 PMC11849266

[ref18] General Office of State Council. (2022). Circular of the general Office of the State Council on further boosting the employment and entrepreneurship of college graduates and other youth. Available online at: https://www.gov.cn/gongbao/content/2022/content_5692853.html

[ref19] Haddoud M. Y. Nowiński W. Laouiti R. Onjewu A.-K. E. (2024). Entrepreneurial implementation intention: the role of psychological capital and entrepreneurship education. Int. J. Manag. Educ. 22:100982. doi: 10.1016/j.ijme.2024.100982

[ref20] Himle J. A. Weaver A. Levine D. S. Steinberger E. Bybee D. Vlnka S. . (2020). Social anxiety and work: a qualitative investigation in a low-income, minority sample. Soc. Work. Ment. Health 18, 302–330. doi: 10.1080/15332985.2020.1742850, PMID: 40746954 PMC12312657

[ref21] Kornblum A. Unger D. Grote G. (2021). How romantic relationships affect individual career goal attainment: a transactive goal dynamics perspective. J. Vocat. Behav. 125:103523. doi: 10.1016/j.jvb.2020.103523

[ref22] Kottwitz M. U. Daibel N. Otto K. (2025). Being pushed or pulled? The role of (in)voluntariness of solo self-employed individuals’ career path in self-fulfillment or precariousness. Adm. Sci. 15:156. doi: 10.3390/admsci15050156

[ref23] Kwak Y. Kim Y. Chae H. (2025). Job search anxiety and flourishing among university students: the serial mediating effects of social support and strengths use. BMC Psychol. 13:652. doi: 10.1186/s40359-025-02995-4 40597452 PMC12211445

[ref24] Lee B.-W. Darmadi D. Gardanova Z. Kostyrin E. Gilmanova N. Kosov M. . (2024). Impact of digital transformation on mental healthcare: opportunities, challenges, and role of AI chat-bots in symptom management. Emerg. Sci. J. 8, 1440–1461. doi: 10.28991/ESJ-2024-08-04-012

[ref25] Lee J. Solomon M. Stead T. Kwon B. Ganti L. (2021). Impact of COVID-19 on the mental health of US college students. BMC Psychol. 9:95. doi: 10.1186/s40359-021-00598-3, PMID: 34103081 PMC8185692

[ref26] Lent R. Brown S. Hackett G. (1994). Toward a unifying social cognitive theory of career and academic interest, choice, and performance. J. Vocat. Behav. 45, 79–122. doi: 10.1006/jvbe.1994.1027

[ref27] Lent R. W. Ireland G. W. Penn L. T. Morris T. R. Sappington R. (2017). Sources of self-efficacy and outcome expectations for career exploration and decision-making: a test of the social cognitive model of career self-management. J. Vocat. Behav. 99, 107–117. doi: 10.1016/j.jvb.2017.01.002

[ref28] Li J. Xue E. Liu B. Han Q. (2024). Impact of COVID-19 on the psychological and behavioral health of college students worldwide: a knowledge mapping approach. Humanit. Soc. Sci. Commun. 11:1353. doi: 10.1057/s41599-024-03781-0

[ref29] Li W. Zhao Z. Chen D. Peng Y. Lu Z. (2022). Prevalence and associated factors of depression and anxiety symptoms among college students: a systematic review and meta-analysis. J. Child Psychol. Psychiatry 63, 1222–1230. doi: 10.1111/jcpp.13606, PMID: 35297041

[ref30] Liu Z. (2025). China’s higher vocational education and training (HVET) graduates’ soft-skill gap: individual aspirations and structural constraints. Res. Post Compuls. Educ. 1–12. doi: 10.1080/13596748.2025.2550847

[ref31] Liu X. Sun X. Hao Q. (2022). Influence of discrimination perception on career exploration of higher vocational students: chain mediating effect test. Front. Psychol. 13:968032. doi: 10.3389/fpsyg.2022.968032, PMID: 35967637 PMC9363697

[ref32] Liu X. Zhang Y. Gao W. Cao X. (2023). Developmental trajectories of depression, anxiety, and stress among college students: a piecewise growth mixture model analysis. Humanit. Soc. Sci. Commun. 10:736. doi: 10.1057/s41599-023-02252-2

[ref33] Lunardon N. Menardi G. Torelli N. (2014). ROSE: a package for binary imbalanced learning. R J. 6, 79–89. doi: 10.32614/RJ-2014-008

[ref34] Matud M. P. (2004). Gender differences in stress and coping styles. Pers. Individ. Differ. 37, 1401–1415. doi: 10.1016/j.paid.2004.01.010

[ref35] Modini M. Joyce S. Mykletun A. Christensen H. Bryant R. A. Mitchell P. . (2016). The mental health benefits of employment: results of a systematic meta-review. Australas. Psychiatry 24, 331–336. doi: 10.1177/1039856215618523, PMID: 26773063

[ref36] Monzonís-Carda I. Rodriguez-Ayllon M. Adelantado-Renau M. Moliner-Urdiales D. (2024). Bidirectional longitudinal associations of mental health with academic performance in adolescents: DADOS study. Pediatr. Res. 95, 1617–1624. doi: 10.1038/s41390-023-02880-z, PMID: 37932488

[ref37] Nation Bureau of Statistics (2017). Industrial classification for national economic activities. Beijing: Former General Administration of Quality Supervision, Inspection and Quarantine National Standardization Administration, 4–86.

[ref38] National Bureau of Statistics. (2022). Announcement of the National Bureau of statistics on the final verification of GDP in 2021. Available online at: https://www.stats.gov.cn/english/PressRelease/202212/t20221227_1891279.html

[ref39] Ranta M. Dietrich J. Salmela-Aro K. (2014). Career and romantic relationship goals and concerns during emerging adulthood. Emerg. Adulthood 2, 17–26. doi: 10.1177/2167696813515852

[ref40] Rivera-Torres P. Araque-Padilla R. A. Montero-Simó M. J. (2013). Job stress across gender: the importance of emotional and intellectual demands and social support in women. Int. J. Environ. Res. Public Health 10, 375–389. doi: 10.3390/ijerph10010375, PMID: 23343989 PMC3564148

[ref41] Seehuus M. Burt K. B. Moeller R. W. (2025). College student mental health is stable across college career, but declining over time. J. Adolesc. 97, 1882–1894. doi: 10.1002/jad.70010, PMID: 40589121 PMC13120353

[ref42] Shao L. Guo H. Yue X. Zhang Z. (2022). Psychological contract, self-efficacy, job stress, and turnover intention: a view of job demand-control-support model. Front. Psychol. 13:868692. doi: 10.3389/fpsyg.2022.868692, PMID: 35602757 PMC9115548

[ref43] Shkoler O. Rabenu E. (2023). The motivations and their conditions which drive students to seek higher education in a foreign country. Curr. Psychol. 42, 25403–25416. doi: 10.1007/s12144-022-03619-5, PMID: 36035251 PMC9395907

[ref44] Siraji A. Molla A. Ayele W. M. Kebede N. (2022). Mental distress and associated factors among college students in Kemisie district, Ethiopia. Sci. Rep. 12, 17541–17548. doi: 10.1038/s41598-022-21710-6, PMID: 36266404 PMC9585086

[ref45] Streiner D. L. (1994). Figuring out factors: the use and misuse of factor analysis. Can. J. Psychiatry 39, 135–140. doi: 10.1177/070674379403900303, PMID: 8033017

[ref46] Thébaud S. (2010). Gender and entrepreneurship as a career choice: do self-assessments of ability matter? Soc. Psychol. Q. 73, 288–304. doi: 10.1177/0190272510377882

[ref47] Tuan T. A. Nghia N. H. An T. D. Loan D. T. T. (2024). Exploring mental stress expressions in online communities: a subreddit analysis. J. Hum. Earth Future 5, 131–150. doi: 10.28991/HEF-2024-05-02-01

[ref48] Vial A. C. Cowgill C. M. (2022). Heavier lies her crown: gendered patterns of leader emotional labor and their downstream effects. Front. Psychol. 13:849566. doi: 10.3389/fpsyg.2022.849566, PMID: 36106035 PMC9465331

[ref49] Walker J. V. Peterson G. W. (2012). Career thoughts, indecision, and depression: implications for mental health assessment in career counseling. J. Career Assess. 20, 497–506. doi: 10.1177/1069072712450010

[ref50] Wiklund J. Yu W. Patzelt H. (2018). Impulsivity and entrepreneurial action. Acad. Manage. Perspect. 32, 379–403. doi: 10.5465/amp.2016.0177

[ref51] Yao K. Zhang E. (2023). China's 2022 economic growth one of the worst on record, post-pandemic policy faces test. Reuters. Available online at: https://www.reuters.com/world/china/chinas-economy-slows-sharply-q4-2022-growth-one-worst-record-2023-01-17/

[ref52] Zhao A. (2023). The impact of career expectation on employment anxiety of art students in higher vocational colleges during the COVID-19: a chain mediating role of social support and psychological capital. Front. Psychol. 14:1141472. doi: 10.3389/fpsyg.2023.1141472, PMID: 36998359 PMC10045042

[ref53] Zhou Y. Liu Y. Xue W. Li X. Yang Z. Xu Z. (2024). Factors that influence the intent to pursue a master’s degree: evidence from Shandong Province, China. Front. Psychol. 15:1284277. doi: 10.3389/fpsyg.2024.1284277, PMID: 38283203 PMC10811022

