# Supplementary material for: Mental health’s shaping influence on college students’ career choices: evidence from L University in Fujian Province, China
Source: Front Psychol. 2025 Dec 16;16:1647718. doi: 10.3389/fpsyg.2025.1647718 (PMC12752113; doi:10.3389/fpsyg.2025.1647718)
Supplement: Supplementary file 1 [file Data_Sheet_1.pdf]

# Supplementary Material

# 大学生心理健康筛查量表

**指导语：**以下列出了一些人可能会有的问题，请仔细阅读每一条，然后根据自己最近一个月的实际感觉，选择最符合您的一种情况，并在答题卡的相应位置上用 2B 铅笔涂好。其中“一点也不像我”涂 1，“不太像我”涂 2，“比较像我”涂 3，“非常像我”涂 4。

| 题号 | 题目 |
|---|---|
| 1 | 不适应大学的生活方式 |
| 2 | 担心找不到好工作 |
| 3 | 感觉学习有困难 |
| 4 | 感觉为恋爱所累 |
| 5 | 不习惯大学班集体的氛围 |
| 6 | 挑剔学校的饮食或住宿条件 |
| 7 | 担心在学习上落后于其他同学 |
| 8 | 感觉不受同学欢迎 |
| 9 | 和班里同学相处不愉快 |
| 10 | 对自己的求职能力没有信心 |
| 11 | 跟周围人有矛盾 |
| 12 | 在恋爱方面受到家人的阻碍 |
| 13 | 总担心自己找不到合适的男/女朋友 |
| 14 | 付出很大努力却仍跟不上学习进度 |
| 15 | 很多时候感觉不被周围人理解 |
| 16 | 担心考试 |
| 17 | 为不知该做哪些就业准备而迷茫 |
| 18 | 害怕面对就业问题 |
| 19 | 谈恋爱对学习、生活造成负面影响 |
| 20 | 不喜欢现在的学校 |
| 21 | 与别人相处时常感到委屈 |
| 22 | 过分在意别人的评价 |
| 23 | 控制不了自己的进食量 |
| 24 | 经常感到别人要欺骗、利用自己 |

| | |
|---|---|
| 25 | 经常无法控制自己激动的情绪 |
| 26 | 顽固地遵守某些规则或习惯，害怕改变 |
| 27 | 故意阻止伤口愈合 |
| 28 | 早上醒来情绪就很低落 |
| 29 | 害怕与人交往 |
| 30 | 经常莫名其妙对别人发火 |
| 31 | 常常自责内疚 |
| 32 | 经常想打人、骂人或毁坏东西 |
| 33 | 身体发麻或刺痛 |
| 34 | 时刻都离不开网络 |
| 35 | 感觉自己在各方面都很失败 |
| 36 | 无法忍受不能上网的生活 |
| 37 | 反应迟钝，注意力不集中 |
| 38 | 有催吐或绝食的行为 |
| 39 | 总是责怪别人造成麻烦 |
| 40 | 感觉自己毫无价值 |
| 41 | 忍不住攻击那些令我不满的人 |
| 42 | 总觉得别人比自己好 |
| 43 | 上网过度影响到正常学习生活 |
| 44 | 没有人安排就不知道该做什么 |
| 45 | 总担心会有不好的事情发生而内心不安 |
| 46 | 总感觉有人在针对我 |
| 47 | 感觉大多数人不可信任 |
| 48 | 总觉得自己不如别人 |
| 49 | 故意抓、戳、咬、撕皮肤至流血 |
| 50 | 常常刚做完一件事就后悔了 |
| 51 | 故意虐待自己 |
| 52 | 经常无缘无故地对别人发火 |
| 53 | 时常因小事而坐立难安 |
| 54 | 凡事需要有人替自己做决定 |
| 55 | 容易被别人的言行伤害 |
| 56 | 睡眠过浅、易醒 |
| 57 | 如果不反复想和做某些事情，就无法继续其他的事 |
| 58 | 做任何事都希望能够获得别人的帮助 |

| | |
|---|---|
| 59 | 常常没考虑清楚就做决定 |
| 60 | 回避与异性交往 |
| 61 | 感觉自己再努力也无法成功 |
| 62 | 故意烧、烫伤自己 |
| 63 | 为脑海中总是抹不去的想法或字句所困扰 |
| 64 | 觉得生活无意义，对任何事都不感兴趣 |
| 65 | 言行常常不考虑后果 |
| 66 | 食欲和进食行为异常，如：吞咽困难、恶心、呕吐等 |
| 67 | 入睡困难或早醒 |
| 68 | 胸闷、呼吸困难 |
| 69 | 与人交谈时过分紧张 |
| 70 | 恶心或胃痛 |
| 71 | 头痛或头晕 |
| 72 | 一旦开始上网就很难停下来 |
| 73 | 没理由地害怕、心神不宁 |
| 74 | 感觉别人都看不起自己 |
| 75 | 在网络上花费了过多的时间 |
| 76 | 别人的言行很容易让我感到受伤 |
| 77 | 经常控制不住自己的手或身体发抖、僵直 |
| 78 | 如果不重复做某件事情，如检查/核对/洗涤或问询，就会觉得很焦虑 |
| 79 | 过度嗜睡 |
| 80 | 避免与别人眼神接触 |
| 81 | 经常和人产生争执 |
| 82 | 对别人都不在意的事情很在意 |
| 83 | 当和别人观点不一致时会感到焦虑 |
| 84 | 做任何事都离不开别人的帮助 |
| 85 | 不敢正视别人的目光 |
| 86 | 过度节食 |
| 87 | 很多事情都无法自己独立处理 |
| 88 | 睡醒后依然感到疲乏 |
| 89 | 能听到旁人听不到的言语声（不是指听力好） |
| 90 | 总感觉有人要害我 |
| 91 | 考虑过自杀的方式或时机 |
| 92 | 想结束生命 |

| | |
|---|---|
| 93 | 曾经有过自杀行为 |
| 94 | 曾经羡慕自杀成功的人 |
| 95 | 总感觉有人在监视我 |
| 96 | 感觉别人能操控我的思想（不是指自身言行受他人言行影响） |

# College Students Mental Health Screening Scale (English version)

**Instruction:** The following are some issues that individuals may experience. Please read each item carefully and choose the option that best describes your feelings in the past month. Mark your answer on the answer sheet using a 2B pencil. Mark “1” for “not at all like me”, “2” for “not very like me”, “3” for “somewhat like me”, “4” for “very much like me”.

| No. | Question |
|---|---|
| 1 | Not adapting to the university lifestyle. |
| 2 | Worried about not finding a good job. |
| 3 | Feeling like learning is difficult. |
| 4 | Feeling burdened by love. |
| 5 | Not used to the atmosphere of the university class collective. |
| 6 | Being picky about the university’s food or accommodation conditions |
| 7 | Worried about falling behind other classmates in studies. |
| 8 | Feeling unwelcome by classmates. |
| 9 | Not getting along with classmates. |
| 10 | Lack confidence in my job-hunting abilities. |
| 11 | Have conflicts with people around you. |
| 12 | Obstructed in love by family. |
| 13 | Always worried about not being able to find a suitable boyfriend/girlfriend. |
| 14 | Put in a lot of effort but still can't keep up with the learning pace. |
| 15 | Many times, I feel misunderstood by the people around me. |
| 16 | Worried about the exam. |
| 17 | Feeling lost about what employment preparations to make. |
| 18 | Afraid to face employment issues. |
| 19 | Dating has a negative impact on studies and life. |
| 20 | I don't like the current university. |
| 21 | Often feel wronged when interacting with others. |
| 22 | Excessively concerned about others' opinions. |
| 23 | Can't control my eating habits. |
| 24 | Often feel that others want to deceive and take advantage of me. |

| | |
|---|---|
| 25 | Often unable to control my excited emotions. |
| 26 | Stubbornly adhering to certain rules or habits, afraid of change. |
| 27 | Deliberately preventing the wound from healing. |
| 28 | Waking up in the morning, I felt very down. |
| 29 | Afraid of socializing with others. |
| 30 | Often inexplicably lose my temper with others. |
| 31 | Often feel self-blame and guilt. |
| 32 | Often want to hit people, curse at them, or destroy things. |
| 33 | Numbness or tingling in the body. |
| 34 | Always inseparable from the internet. |
| 35 | I feel like I'm failing in every aspect. |
| 36 | Unable to endure a life without the internet. |
| 37 | Slow reaction, lack of concentration. |
| 38 | Engaging in behaviors such as inducing vomiting or fasting. |
| 39 | Always blaming others for causing trouble. |
| 40 | Feeling completely worthless. |
| 41 | Can't help but attack those who displease me. |
| 42 | Always feel like others are better than me. |
| 43 | Excessive internet use affects normal study and life. |
| 44 | If no one makes arrangements, we won't know what to do. |
| 45 | Always worried that something bad will happen, feeling uneasy inside. |
| 46 | I always feel like someone is targeting me. |
| 47 | I feel like most people can't be trusted. |
| 48 | Always feel like I'm not as good as others. |
| 49 | Deliberately scratch, poke, bite, and tear the skin until it bleeds. |
| 50 | Often, I regret something right after finishing it. |
| 51 | Deliberately self-harming. |
| 52 | Often gets angry at others for no reason. |
| 53 | Often restless over trivial matters. |
| 54 | Everything requires someone to make decisions for you. |
| 55 | Easily hurt by others' words and actions. |
| 56 | Light sleep, easily awakened. |
| 57 | If you don't repeatedly think about and do certain things, you won't be able to continue with other things. |

| | |
|---|---|
| 58 | In everything I do, I hope to receive help from others. |
| 59 | Often make decisions without thinking them through. |
| 60 | Avoid interacting with the opposite sex. |
| 61 | Feels like no matter how hard I try, I can't succeed. |
| 62 | Deliberately burning or scalding oneself. |
| 63 | Troubled by thoughts or phrases that can never be erased from the mind. |
| 64 | Feel that life is meaningless and not interested in anything. |
| 65 | Words and actions often do not consider the consequences. |
| 66 | Abnormal appetite and eating behaviors, such as difficulty swallowing, nausea, vomiting, etc. |
| 67 | Difficulty falling asleep or waking up early. |
| 68 | Chest tightness, difficulty breathing. |
| 69 | Too nervous when talking to people. |
| 70 | Nausea or stomach pain. |
| 71 | Headache or dizziness. |
| 72 | Once you start browsing the internet, it's hard to stop. |
| 73 | Fear and anxiety without reason. |
| 74 | Feel like everyone looks down on me. |
| 75 | Spent too much time online. |
| 76 | Other people's words and actions can easily hurt me. |
| 77 | Often unable to control their hands or body, trembling or stiffening. |
| 78 | If I don't repeatedly do something, like checking, verifying, washing, or inquiring, I feel very anxious. |
| 79 | Excessive sleepiness. |
| 80 | Avoid making eye contact with others. |
| 81 | Often gets into arguments with people. |
| 82 | Things that others don't care about, I care a lot about. |
| 83 | When I have a different opinion from others, I feel anxious. |
| 84 | Doing anything is inseparable from the help of others. |
| 85 | Dare not face others’ gazes. |
| 86 | Excessive dieting. |
| 87 | Many things cannot be handled independently. |
| 88 | Still feeling tired after waking up. |
| 89 | Can hear voices that others can't (not referring to good hearing). |
| 90 | I always feel like someone is trying to harm me. |

| | |
|---|---|
| 91 | Considered the method or timing of suicide. |
| 92 | Want to end my life. |
| 93 | Had a history of suicidal behavior. |
| 94 | Once envied those who succeeded in suicide. |
| 95 | I always feel like someone is watching me. |
| 96 | I feel like others can control my thoughts (not referring to my own words and actions being influenced by others). |
